# Clinical and histopathological correlation of oral malignancy and potentially malignant disorders based on a screening program at high-risk population in Tamil Nadu, India

**DOI:** 10.3389/froh.2023.1286780

**Published:** 2023-11-03

**Authors:** Kiran Iyer, Madan Kumar, Ranganathan Kannan, Aswath Narayanan, Muhamood Moothedath, Sanjeev Balappa Khanagar, Laliytha Kumar Bijai

**Affiliations:** ^1^ Dental Public Health, Preventive Dental Science, College of Dentistry, King Saud bin Abdulaziz University for Health Sciences, King Abdullah International Medical Research Centre, Riyadh, Saudi Arabia; ^2^ Department of Public Health Dentistry, Ragas Dental College and Hospital, Chennai, India; ^3^ Department of Oral and Maxillofacial Pathology, Ragas Dental College and Hospital, Chennai, India; ^4^ The Tamil Nadu Dr. M.G.R Medical University, Chennai, India; ^5^ Department of Oral and Dental Health Sciences in Ar Rass, Qassim University, Al Qassim, Saudi Arabia; ^6^ Oral Medicine and Maxillofacial Radiology, Maxillofacial Surgery and Diagnostic Sciences, College of Dentistry, King Saud bin Abdulaziz University for Health Sciences, King Abdullah International Medical Research Centre, Riyadh, Saudi Arabia

**Keywords:** high risk population, oral cancer, oral potentially malignant disorders, screening, tobacco

## Abstract

**Background:**

There is a high incidence of oral cancer and oral potential malignant disorder observed in southeast Asian countries such as India. Our study aimed to assess the correlation between screening and histopathological diagnosis and to predict the specificity and sensitivity of chair-side/field-based assessment of the oral lesion.

**Materials and methods:**

A total of 40,852 subjects aged between 20 and 60 years were screened in the 1st phase of the study, suspected lesions were stained with toluidine blue (Manufactured by Otto Chemicals private limited, India) at two time points, those who stained positively during the two points were taken up for biopsy. Provisional diagnosis was later correlated with histopathological diagnosis.

**Results:**

Subjects who underwent biopsy had a mean age of (49.01 ± 9.8 years), Leukoplakia (1.5%) was the most common lesion observed among tobacco users, interestingly it had the least correlation (39.6%) in diagnosis, Overall sensitivity (88%) and a positive predictive value (80%) was high for clinical diagnosis of OPMD in our study.

**Conclusion:**

Correlation of clinical and histopathological diagnosis observed in our study confirms higher yield of true positives while screening in remote and vulnerable populations, which would assure a better quality of life for these subjects.

## 1. Introduction

India witnesses one-third of the global oral cancer cases, with an incidence of one-fourth of the global mortality due to oral cancer. A high percentage of these cases are identified and diagnosed at the advanced stage of the disease. Early detection of potentially malignant lesions ensures a better prognosis and quality of life (1–3).

**Figure 1:**
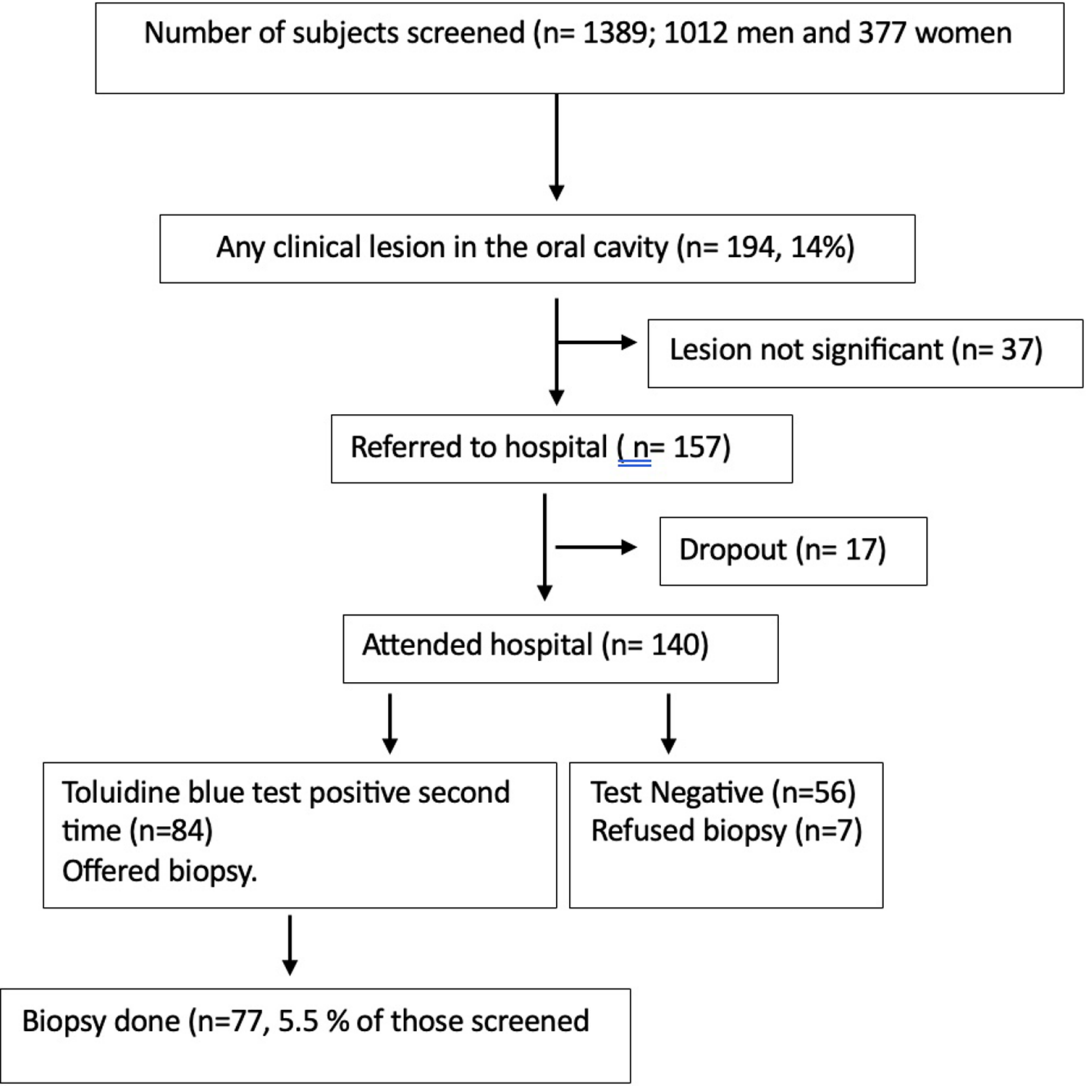
Flow chart on the details of participants screened, diagnosed clinically, and referred for biopsy and biopsied was presented.

The increased incidence of oral cancer is of alarming concern to public health and dental public health, as this has emerged over the years to be the most common type of cancer in the country, this is despite various regulatory measures undertaken by the country to create awareness and restrict exposure to the associated risk factors (4).

Screening has been an effective approach, to identifying asymptomatic cases, with varying degrees of success, leading to questioning of the feasibility (2). Risk-based screening has been proven to have greater efficiency in comparison with general population-based screening as evident from the literature. These screening procedures become more resourcefully feasible if healthcare workers (HCWs) are enrolled (after appropriate training and calibration) to carry out the initial screening of the oral cavity and refer the potential cases for investigation to hospitals (5, 6).

Though the histopathological investigation is the gold standard for confirming the diagnosis, the emphasis lies on appropriate referral based on thorough clinical assessment of the oral cavity and the lesion when provisionally identified, when executed with acumen by the clinician or trained health care workers saves resources and unwarranted invasive intervention among cases (7).

There is a lacuna in the literature concerning the correlation between the screening-based diagnosis of oral cancer or other potentially malignant lesions with the histopathological diagnosis, few studies in the past have attempted to ascertain this correlation with retrospective data (8, 9). This correlation based on the sensitivity and specificity of chair-side diagnosis becomes important to help standardize the diagnostic technique used for screening, leading to uniform reporting and appropriate use of limited resources. The study had various primary objectives which have been analyzed and published in the past, this paper was taken up with the intention to analyze and discuss the sensitivity and specificity of the visual oral screening when compared to the histological findings, which were considered the “gold standard” (10).

The present study was undertaken among a large population of “high-risk” individuals at Ranipet industrial town of Tamil Nadu, India. The study aimed to assess the correlation between screening and histopathological diagnosis, to predict the specificity and sensitivity of chair-side/field-based assessment of the oral lesion.

## 2. Materials and methods

Ranipet a district in the Tamil Nadu state of India, with the namesake town is a semi-urban region composed of a heterogeneous population. It serves as an industrial hub, with a thriving tannery industry. There is a high prevalence of tobacco and alcohol use observed in the population.

In the present population-based screening study a memorandum of understanding (MOU) was drafted and agreed upon between Thirumalai mission trust hospital located in Vanapadi village in Ranipet district and ragas dental college and hospital, Chennai. The trust hospital caters to the healthcare needs of approximately 142,150 people (315 villages and 35,000 families) and has been doing so for more than a decade.

Awareness programs are undertaken by the trust-based hospital in this population with an organized and trained workforce consisting of family care volunteers (FCV) for approximately every 50 households working under the supervision of multipurpose workers (MPWs) (one for about 500–1,000 households). FCV are well acquainted with the communities and are part of the community, hence involving them ensured better participation and understanding among the screened population. Based on this local manpower availability, and technical and infrastructural assistance of the trust-based hospital an oral cancer screening program was conceptualized between the hospital and the dental college. The screening (cross-sectional) of the whole population in the region was carried out between August 2018 and December 2019. Ethical clearance was obtained from the Institutional Review Board of the hospital and the dental college (project 20180703 approved on July 30, 2018) before the commencement of the screening program. The findings of this study have been reported by STROBE guidelines (11).

Written informed consent was obtained from all study participants prior to enrolling them in the study, they were made aware of all the aspects of participation and outcomes, and the information was conveyed in the vernacular language (Tamil) for better understanding and compliance. The study was conducted according to the ethical guidelines established by the Declaration of Helsinki and other guidelines like Good Clinical Practice Guidelines and those established by the Indian Council for Medical Research.

Adult participants irrespective of oral adverse habits were considered and were initially screened for the presence of oral lesions. Of the total population considered for the study 71,356 people aged between 21 and 60 years were deemed to be enrolled in the study. The coverage area was divided into two zones (ZONE I AND ZONE II) for the convenience of investigators, these zones had a population of 40,852 and 30,504 respectively. A 1:2 ratio of the case to control was adopted in the study. All known potential confounders such as age, gender, habits and occupation were matched between the cases and controls. Controls were selected from the same population without any history of oral adverse habits.

The intra-oral assessment was carried out by the dentists in the field setting (at anganwadis and schools in the vicinity of the population screened). American Dental Association (ADA) oral examination classification III was used for assessment in field settings with the artificial light source. While screening, if a potential lesion was identified, a subject expert's opinion was sorted by sending images through the mobile application (WhatsApp). Variations of the normal mucosa and certain non-significant pathological changes of mucosa were not further investigated (usual variants of mucosal keratosis, smokers' palate, denture stomatitis, etc.). Suspected lesions were taken up for staining during two time periods, firstly at the field setting, those lesions which positively stained with toluidine blue, these patients were asked to visit the dental clinic at the hospital for the second, those lesions which positively stained on both occasions were further taken up for biopsy (5 mm punch biopsy), biopsy sample collection was done by a trained dentist. The sample was stored in 10% formaldehyde, transported within 3–4 h, and was sent to oral pathology department of ragas dental college and hospital for histopathologic examination. Sutures were placed at the intra-oral site of biopsy collection in patients who underwent the procedure, and after a week suture removal was undertaken, ensuring proper healing at the site.

All subjects with a history of tobacco use underwent counseling at de-addiction center of the hospital. Nicotine replacement therapy to overcome tobacco use was given free of cost as part of the de-addiction and counseling process.

Those identified with established oral malignant lesions were referred to a regional cancer care center (Aringar Anna Cancer Treatment Center, Kanchipuram, Tamil Nadu, India) for appropriate treatment and follow-up. The data were cleaned and entered in Microsoft Excel for analysis. Descriptive analysis was carried out for to express the frequencies. Sensitivity and specificity of the diagnosis during initial screening was assessed against the histopathological gold standard. Statistical Package for Social Services (Version 20.0 for Windows, SPSS Inc., Chicago, IL, USA) and Microsoft Excel was used for statistical analysis.

## 3. Results

The screening program results have already been presented in an earlier publication by the authors (10). In this screening program, 77 biopsies were taken from subjects, among whom 74 were histologically examined. Three specimens were excluded as they were found to be inadequate by the pathologist.

Table 1 depicts variables of interest such as total population screened 40,852 (28.7%), patients who underwent biopsy had a mean age of (49.01 ± 9.8 years), tobacco exposure (15.2 ± 11.9 years), and an exposure factor of (173.80 ± 213.6). Leukoplakia (1.5%) was the most common OPMD observed in the tobacco users and left buccal mucosa (36.4%) was the most common site of OPMD occurrence.

**Table 1:** Variables of interest in the screened population.

Variables of interest	Observation
Total Population screened	40,852 (28.7%) of 142,150
Mean age of participants who underwent biopsy	49.01 ± 9.8 years
Mean years of tobacco exposure in biopsy patients	15.2 ± 11.9 years
Mean exposure factor	173.80 ± 213.6 (Usage per day multiplied by the number of years of usage)
Most common OPMD: Leukoplakia	21/41 (51.2%) of all OPMD 21/1389 (1.5%) of all Tobacco users
Most common site of OPMD	27 (36.4%) Left buccal mucosa

Table 2 demonstrates the clinical vs. the histological diagnosis for the biopsy specimen. Among 43 (58.1%) subjects with a clinical diagnosis of Leucoplakia, only 21 were confirmed to have dysplastic changes during the histological examination. Except for those diagnosed with fibroma and frictional keratosis (Histologically confirmed as hyperkeratosis with acanthosis), histologically dysplastic features were confirmed for those subjects with a clinical diagnosis of potentially malignant oral lesions.

**Table 2:** Frequency correlation of clinical and histopathological diagnosis of OPMD.

Clinical diagnosis	Histopathological diagnosis						
	Mild dysplasia	Moderate dysplasia	Oral sub-mucous fibrosis	Hyper-keratosis with acanthosis	Epithelial atypia	Lichenoid mucositis	Total
Leucoplakia	16	1	4	17	3	2	43 (58.1%)
Erythro-leukoplakia	3	–	–	1	–	2	6 (8.1%)
Verrucous leucoplakia	2	–	–	–	–	–	2 (2.7%)
Oral submucous fibrosis	1	–	9	–	–	–	10 (13.5%)
Fibroma	–	–	–	2	–	–	2 (2.7%)
Tobacco pouch keratosis	5	–	–	4	–	–	9 (12.1%)
Lichen planus	1	–	–	–	–	–	1 (1.4%)
Frictional keratosis	-	–	–	1	–	–	1 (1.4%)
Total	28 (37.8%)	1 (1.4%)	13 (17.6%)	25 (33.8%)	3 (4.1%)	4 (5.4%)	74 (100%)

Though the overall sensitivity of clinical diagnosis was 88%, the sensitivity values ranged from 39.6% for Leucoplakia to 100% for Verrucous leukoplakia and Lichen planus. However, the positive predictive value was over 80% for all the clinical conditions recorded in the screening program (Table 3).

**Table 3:** Sensitivity and positive predictive value (PPV) in relation to clinical diagnosis of OPMD as observed in the study.

Clinical diagnosis	Sensitivity	Positive predictive value
Leukoplakia	39.6%	80.9%
Erythroleucoplakia	50%	100%
Verrucous leukoplakia	100%	100%
Oral submucous fibrosis	90%	90%
Tobacco pouch keratosis	55.5%	100%
Lichen planus	100%	100%

## 4. Discussion

Evidence suggests that early intervention among the identified true positive cases have a good prognosis, improved survival rate and better quality of life than otherwise (12, 13). A recent systematic review outlines the significance of high-risk population-based screening which could save “two to three times more lives than non-targeted screening” (14, 15). In the present study, a high-risk population in an industrial town was considered for screening.

The mean age of patients referred for biopsy in our study was 49.01 ± 9.8 which is in line with study by Torabi et al. (15), other studies on the similar topic have reported aa slightly higher mean age of 56 years and 55 years by Maia et al, and Mehrotra et al., respectively.

Leukoplakia was the most common OPMD observed in our screening population, similar finding has been reported on leukoplakia in studies by Chher et al., and Pentenero et al. (16, 17), who undertook oral mucosal lesion assessment in large population screening at Cambodia and Italy respectively. Contrastingly studies by Oivio et al. (18), and Feng et al. (19), in Finland and China have reported oral lichen planus to be the major OPMD diagnosed in their screening population.

Left buccal mucosa was the most common site of OPMD followed by right buccal mucosa and right vestibule in our study, the finding of buccal mucosa to be the most common site of OPMD is in line with studies by Torabi et al. (15), and M. Bokor-Bratic et al., (20). Recently the WHO Collaborating Centre for oral cancer comprising the expert group on oral cancer and OPMD revised the classification of OPMDs and considered Oral leukoplakia, oral lichen planus, oral lichenoid lesions, proliferative verrucous leukoplakia, and oral submucous fibrosis as OPMDs (15). These OPMDs have a differing percentage of malignant transformation based on the type of lesion from 5%–18%.

The present study observed an average 15.2 years of habit (smoke, smokeless tobacco, betel quid, and areca nut) prevalence among those identified for biopsy, and a steady increase in OPMD cases was observed when average usage per day was ascertained (from <5 to >20). These observations are in line with study by Shivakumar et al. (21), who reported 7.31 ± 6.94 years of habit (tobacco) prevalence in OPMD cases and average usage per day at 4.92 ± 4.02.

Exposure factor (usage per day multiplied by the number of years of usage) also indicated an increased prevalence of OPMD with increase in the exposure factor. The average exposure factor of 173.80 was observed in the present study. This variable in our study is in concordance with person years observation of subjects with adverse oral habits in a study by Sankaranarayanan et al. (22), who screened for oral cancer cases in Trivandrum district of Kerala, India and reported increased incidence and mortality as person years of habit observations increased between cases and controls.

There was an inverse correlation observed with respect to area of lesion in our study, most of the lesions which measured (1–1,000 sq mm) were non OPMD lesions. This finding is interesting and has not been reported previous in literature. As well as there was no significantly different consistency observed between OPMD (33 soft and 8 firm) and non-OPMD lesions (27 soft and 6 firm).

Screening related sensitivity for OPMD was found to be good in the present study at 88%, various studies have reported on the similar lines while assessing the concurrence of screening with histopathological diagnosis. Study on clinicopathologic correlation of white lesions by Abidullah et al. (23), reported a 78% correlation.

The highest correlation in clinicopathological diagnosis was observed for lichen planus (100%) and Verrucous Leukoplakia (100%) followed by oral submucous fibrosis (90%). Interestingly though leukoplakia was the most common OPMD only 39.6% sensitivity was observed while diagnosing it clinically. This may be since leukoplakia being a clinical term does not specifically identify with the histopathological presentation, the degree of lesion presentation with that of histopathology is low. In the present study, clinical and histopathological correlation were inconsistent with erythroleukoplakia cases too, these has been correlated to similarity in presentation of severe dysplasia and carcinoma *in situ*.

There is a need for sustained oral cancer screening as part of national health survey, Taiwan is the only country in the world that has implemented a sustained oral cancer screening program, and it should be noted that it currently offers screening to high-risk patients (betel chewers or former betel chewers and smokers) (2). These screening procedures would be beneficial especially in the south-east Asian regions, where OPMD prevalence is found to be very high.

Another aspect that needs attention to increase the correlation in diagnosis and the yield of screening is to standardize training of dentists and their examiners, this variability of training hinders clinical diagnosis.

This study has added strength to exiting data on correlation of clinical and histopathological diagnosis of OPMD, a high sensitivity was observed for visual screening of lesions and hence can serve as an efficient tool in initial screening especially at remote and high-risk population. The findings of these study have certain limitations such as attrition of patients when referred for biopsy, which might be because of anxiety, insufficient biopsy sample for certain cases which was eventually not included as part of the study. Further studies can be undertaken with caution on these limitations.

## Data Availability

The raw data supporting the conclusions of this article will be made available by the authors, without undue reservation.
